# The serum-based VeriStrat® test is associated with proinflammatory reactants and clinical outcome in non-small cell lung cancer patients

**DOI:** 10.1186/s12885-018-4193-0

**Published:** 2018-03-20

**Authors:** Mary Jo Fidler, Cristina L. Fhied, Joanna Roder, Sanjib Basu, Selina Sayidine, Ibtihaj Fughhi, Mark Pool, Marta Batus, Philip Bonomi, Jeffrey A. Borgia

**Affiliations:** 1 Sections of Medical Oncology at Rush University Medical Center, Chicago, USA; 2 Pathology, Rush University Medical Center, Chicago, USA; 3 Biodesix, Inc, Boulder, CO 80301 USA; 4 Preventative Medicine, Rush University Medical Center, Chicago, USA; 5 Cell and Molecular Medicine at Rush University Medical Center, Il, Chicago, 60612 USA; 6 Departments of Pathology and Cell & Molecular Medicine, Rush University Medical Center, 570 Jelke Southcenter Bldg.,1750 W. Harrison St, Chicago, IL 60612 USA

**Keywords:** Biomarker, Serum, Non-small cell lung cancer (NSCLC), Erlotinib, Luminex, VeriStrat

## Abstract

**Background:**

The VeriStrat test is a serum proteomic signature originally discovered in non-responders to second line gefitinib treatment and subsequently used to predict differential benefit from erlotinib versus chemotherapy in previously treated advanced non-small cell lung cancer (NSCLC). Multiple studies highlight the clinical utility of the VeriStrat test, however, the mechanistic connection between VeriStrat-poor classification and poor prognosis in untreated and previously treated patients is still an active area of research. The aim of this study was to correlate VeriStrat status with other circulating biomarkers in advanced NSCLC patients – each with respect to clinical outcomes.

**Methods:**

Serum samples were prospectively collected from 57 patients receiving salvage chemotherapy and 70 non-EGFR mutated patients receiving erlotinib. Patients were classified as either VeriStrat good or poor based on the VeriStrat test. Luminex immunoassays were used to measure circulating levels of 102 distinct biomarkers implicated in tumor aggressiveness and treatment resistance. A Cox PH model was used to evaluate associations between biomarker levels and clinical outcome, whereas the association of VeriStrat classifications with biomarker levels was assessed via the Mann-Whitney Rank Sum test.

**Results:**

VeriStrat was prognostic for outcome within the erlotinib treated patients (HR = 0.29, *p* < 0.0001) and predictive of differential treatment benefit between erlotinib and chemotherapy ((interaction HR = 0.25; interaction *p* = 0.0035). A total of 27 biomarkers out of 102 unique analytes were found to be significantly associated with OS (Cox PH *p* ≤ 0.05), whereas 16 biomarkers were found to be associated with PFS. Thrombospondin-2, C-reactive protein, TNF-receptor I, and placental growth factor were the analytes most highly associated with OS, all with Cox PH *p*-values ≤0.0001. VeriStrat status was found to be significantly associated with 23 circulating biomarkers (Mann-Whitney Rank Sum p ≤ 0.05), 6 of which had *p* < 0.001, including C-reactive protein, IL-6, serum amyloid A, CYFRA 21.1, IGF-II, osteopontin, and ferritin.

**Conclusions:**

Strong associations were observed between survival and VeriStrat classifications as well as select circulating biomarkers associated with fibrosis, inflammation, and acute phase reactants as part of this study. The associations between these biomarkers and VeriStrat classification might have therapeutic implications for poor prognosis NSCLC patients, particularly with new immunotherapeutic treatment options.

**Electronic supplementary material:**

The online version of this article (10.1186/s12885-018-4193-0) contains supplementary material, which is available to authorized users.

## Background

The VeriStrat (VS) test classifies patients as either good or poor based on a matrix assisted laser desorption/ionization time-of-flight (MALDI-TOF) mass spectrometry protein signature [1]. It has been shown to be prognostic for outcomes in advanced NSCLC patients treated with EGFR TKIs and platinum-based chemotherapy and predictive of differential survival benefit between EGFR TKIs and single agent chemotherapy [1–6].

The prognostic benefit of the VS test has been demonstrated with other therapies for NSCLC, including those targeting angiogenic pathways. Analysis of cohorts treated with combinations erlotinib and bevacizumab or erlotinib and sorafenib showed superior overall survival for the good classification group compared with the poor group [7–11]. In addition, studies of VS in patients treated with front line platinum doublet chemotherapy [12, 13] and in previously treated patients receiving nivolumab [14] indicate that VS’s prognostic ability extends to other therapeutic regimens.

Multiple isoforms of serum amyloid A contribute to the 8-peak proteomic signature that underpins the VS test, but the identity of some of the other components of the signature remain unknown [1, 15]. As expression of serum amyloid A, an acute phase protein, is known to play a role in the VS test classification, it is to be expected that the VeriStrat classification is associated with other proteins related to the acute response and/or chronic inflammation, as well.

The objective of this study was to evaluate potential correlations between VS good and poor classifications, outcomes, and circulating biomarkers implicated in tumor progression and treatment resistance in pretreatment sera collected from advanced NSCLC patients treated with second line cytotoxic chemotherapy or erlotinib.

## Methods

### Patient population

The Rush University Medical Center (RUMC) biorepository houses biospecimens (serum, plasma, plasma buffy coats) from over 500 cases of advanced stage NSCLC. From this cohort, we selected cases that failed front-line treatment and were treated with either cytotoxic agents or erlotinib. Individual treatments were selected by the physician in accordance to standards of care. Disease progression were assigned to all cases based on version 1.1 of RECIST criteria. Serum and clinical data were collected prospectively with written informed patient consent. This study was reviewed and approved by the Institutional Review Board at RUMC.

### Collection and storage of serum specimens

Peripheral blood was collected in standard 10 mL red-top Vacutainers® from each patient immediately prior to treatment initiation. Samples were processed using standard phlebotomy methods, as previously described [16]. A portion of each serum sample used for the Luminex evaluations were supplemented with 25 μL/mL of the Mammalian Protease inhibitor cocktail (Sigma, St. Louis, MO) and 10 μL/mL of 0.5 M EDTA to minimize further proteolysis. Aliquots were archived in a-80 °C freezer until testing. No specimen tested in this study was subject to greater than two freeze-thaw cycles.

### EGFR mutational status

EGFR mutational status was determined when possible from archival FFPE materials as we previously described [17]. When FFPE material was not available, digital droplet PCR was used to determine mutational status on cell-fee DNA in patient plasma, also as previously described [16]. Briefly, circulating free DNA (cfDNA) was purified from plasma (yellow top - ACD) using the NucleoSpin Plasma XS kit (Clontech Laboratories) and evaluated on a NanoDrop (Agilent Technolgies, Santa Clara, CA) or Qubit (ThermoFisher Scientific) spectrophotometer. A Bio-Rad QX200 digital PCR System (Bio-Rad Laboratories) was then used to interrogate the specimens for the EGFR mutations G719S and L858R as well as an exon 19 deletion (E746-A750). Amplicon levels were determined on a QX200 Droplet Reader and analyzed using the QuantaSoftTM software (Bio-Rad).

### Measurements of serum biomarker levels

Serum specimens were evaluated with a total of 104 assays (consisting of 102 unique analytes), performed using Luminex immunobead assays as indicated below. All primary data points were collected on a Luminex FLEXMAP 3D® system. Analyte concentrations were calculated from a 7-point curve using a five-parametric fit algorithm (xPONENT® v4.0.3 Luminex Corp., Austin, TX). All data met minimum quality control thresholds defined by the kit manufacturer with percent coefficient of variation (%CV) values ≤10%, all as previously defined [16].

Biomarkers used in the current study were as follows: IGF-I (MILLIPLEX® MAP Human IGF-I Single Plex; EMD Millipore Corp., Billerica, MA), IGF-II (MILLIPLEX® MAP Cancer Biomarker Panel; EMD Millipore Corp., Billerica, MA), IGFBP-1, IGFBP-2, IGFBP-3, IGFBP-4, IGFBP-5, IGFBP-6, IGFBP-7 (MILLIPLEX® MAP Human IGF Binding Protein (IGFBP) Panel; EMD Millipore Corp., Billerica, MA), angiopoietin-2, G-CSF, BMP-9, endoglin, endothelin-1, FGF-1, follistatin, IL-8, HGF, HB-EGF, PLGF, VEGF-C, VEGF-D, FGF-2, VEGF-A (MILLIPLEX® MAP Human Human Angiogenesis/ Growth Factor Panel 1; EMD Millipore Corp., Billerica, MA), angiostatin, sAXL, sc-kit/SCFR, sHer2, sHer3, sE-selectin, sHGFR/c-Met, tenascin-C, PDGF-AB/BB, sIL-6Ralpha, sTie-2, thrombospondin-2, sNeuropilin-1, sEGFR, suPAR, sVEGFR1, sVEGFR2, sVEGFR3, sPECAM-1 (MILLIPLEX® MAP Human Osteopontin Human Angiogenesis Panel 2; EMD Millipore Corp., Billerica, MA), sEGFR, sCD30, sgp130, sIL-1RI, sIL-1RII, sIL-2Ralpha, sIL-4R, sIL-6R, sRAGE, sTNFRI, sTNFRII, sVEGFR1, sVEGFR2, sVEGFR3 (MILLIPLEX® MAP Human Soluble Cytokine Receptor Panel; EMD Millipore Corp., Billerica, MA), HCG, α-fetoprotein, CA-125, CA 15–3, CA 19–9, CEA, HE4, MIF, osteopontin, prolactin, SCF, sFas, sFasL, TGF-α, TNF-α, total PSA, TRAIL, CYFRA 21-1 (MILLIPLEX® MAP Human Circulating Cancer Biomarker Panel 1) amphiregulin, betacellulin, epiregulin, EGF, HB-EGF, PDGF-BB, PLGF, tenascin C (Widescreen Human Cancer Panel 2, EMD Millipore Corp.), adipsin and adiponectin (Human Diabetes 2-plex; Bio-Rad Laboratories, Inc., Hercules, CA), insulin, GIP, glucagon, visfatin, ghrelin, GLP-1, PAI-1, resistin, C-peptide, leptin (Human Diabetes 10-plex; Bio-Rad Laboratories, Inc., Hercules, CA), haptoglobin, CRP, alpha-2- macroglobulin, serum amyloid P, tissue plasminogen activator, ferritin, fibrinogen, procalcitonin, serum amyloid A (Human Acute Phase 5 + 4-plex Panel; Bio-Rad Laboratories, Inc., Hercules, CA).

### VeriStrat classifications

VeriStrat (VS) testing was performed as described [1, 3]. The test is based on MALDI mass spectrometry (MS). All samples were provided to Biodesix and processed in a blinded manner; only Rush investigators had access to information beyond specimen code at the time of testing. Ion current (intensity) values of eight spectral regions were evaluated in triplicate and compared to a standard reference set in order to assign a good or poor classification label. An indeterminate classification status was assigned to cases with discordant findings in the replicates. Only patients with classifications of VeriStrat good (VSG) or VeriStrat poor (VSP) were included in the study cohort.

### Biomarker statistical methods

The erlotinib and chemotherapy groups were evaluated for differences between clinic-demographic parameters using the Mann-Whitney and Fisher’s exact tests. Time-to-event outcomes (PFS/OS) were associated with biomarkers concentrations in a continuous scale using the Cox proportional hazards (PH) regression analyses. The association of VeriStrat classification with treatment grouping and progression-free survival (PFS) and overall survival (OS) were accomplished using the multivariate Cox PH interaction model, in a manner similar to other studies [6].

The association of VS status with circulating biomarker levels was evaluated with the Mann-Whitney Rank Sum test and graphically reported as box-and-whisker plots. False discovery rate (FDR) was calculated for association of biomarker concentrations with outcomes and VeriStrat classification using the method of Benjamini and Hochberg [18].

## Results

### Patient demographics and clinical correlates

This prospective non-randomized study included a cohort of advanced NSCLC patients from RUMC who had disease progression on front-line platinum doublet based chemotherapy and were treated subsequently with either cytotoxic agents (*n* = 57) or erlotinib (*n* = 70). Treatment was chosen at the discretion of the patient’s physician. The study cohort was 53% female, 72% white, with 87% smokers. Median age was 65 years and 63% had performance status 1 and 80% of patients had non-squamous disease. No statistically significant differences in population with respect to patient characteristics were detected between the two treatment cohorts (Table 1). Briefly, the mean age was 64.0 years for both sub-cohorts, while gender distributions were 49.2% and 55.7% female for chemotherapy and erlotinib arms, respectively. As shown in Table 1, the gender difference was not statistically different. Racial distributions were also similar between the chemotherapy and erlotinib cohorts, consisting primarily of white subjects (73.0% and 74.3%, respectively), black (26.3% and 21.4%, respectively), with the balance being Asian or Asian/ Pacific Islanders. Both arms were composed chiefly of non-squamous histology (79.0% chemotherapy, 81.4% erlotinib), and this difference was not statistically significant (*p* = 0.8235). An overwhelming majority of the subjects in both cohorts were current or former smokers, with a slightly higher portion of which in the chemotherapy cohort (91.2% versus 82.9%, chemotherapy and erlotinib; *p* = 0.0831). EGFR mutation status was evaluated in 77% of the chemotherapy cohort and 63% of the erlotinib cohort, when evaluable specimens (tumor or plasma) were available; however no EGFR mutations were detected in any sample.

**Table 1:** Patient characteristics by treatment type

	Chemotherapy (*n* = 57)	erlotinib (*n* = 70)	*p* value
Age			
Mean (SD)	64.0 (8.9)	64.0 (9.7)	0.9960
Median (Range)	65.1 (44.2–83.7)	64.5 (40.9–88.2)	0.8383
VeriStrat Classification, n (%)			0.6865
Good	41 (71.9)	53 (75.7)	
Poor	16 (28.1)	17 (24.3)	
Gender, n (%)			0.4800
Female	28 (49.2)	39 (55.7)	
Male	29 (50.9)	31 (44.3)	
Race			0.2711
White	40 (70.2)	52 (74.3)	
Black	16 (28.1)	15 (21.4)	
Asian/Pacific Islander	0 (0)	3 (4.3)	
Asian	1 (1.8)	0 (0)	
Histology, n (%)			0.1845
Adenocarcinoma	34 (59.6)	46 (65.7)	
Adenosquamous	2 (3.5)	0 (0)	
Bronchioalveolar	0 (0)	1 (1.4)	
Bronchogenic carcinoma	1 (1.8)	0 (0)	
Carcinoma	6 (10.5)	9 (12.9)	
Large Cell	0 (0)	1 (1.4)	
NSCLC	3 (5.3)	0 (0)	
Neuroendocrine	1 (1.8)	0 (0)	
Squamous	10 (17.5)	13 (18.6)	
Smoking Status, n (%)			0.0831
Yes	52 (91.2)	58 (82.9)	
No	4 (7.0)	12 (17.1)	
Missing	1 (1.8)	0 (0)	
Performance Status, n (%)			0.6697
0	12 (21.1)	16 (22.9)	
0.5	1 (1.8)	0 (0)	
1	35 (61.4)	45 (64.3)	
1.5	1 (1.8)	1 (1.4)	
2	8 (13.1)	6 (8.6)	
3	0 (0)	2 (2.9)	
Grade n (%)			0.4455
Moderately	6 (10.5)	10 (14.3)	
Moderately/Poorly	2 (3.5)	3 (4.3)	
Nos	26 (45.6)	26 (37.1)	
Poorly	21 (36.8)	25 (35.7)	
Well	1 (1.8)	6 (8.6)	
Well/Moderately	1 (1.8)	0 (0)	

### VeriStrat status and associations with PFS and OS at RUMC

VS labels were similarly distributed in both treatment cohorts; 72% of the chemotherapy and 76% of the erlotinib cohort were classified as VeriStrat good (VSG) (*p* = 0.6865) (Table 1). Further, VeriStrat classification was independent of age, gender and racial distributions, smoking status, and tumor histology/grade (*p* > 0.10). Patient characteristics with respect to VS status are provided as Additional file 1: Table S1. Not surprisingly, there was a trend towards (*p* = 0.0807) a superior performance status in the VSG group relative to those classified as VeriStrat poor (VSP), as shown in Additional file 2: Table S2.

**Fig. 1:**
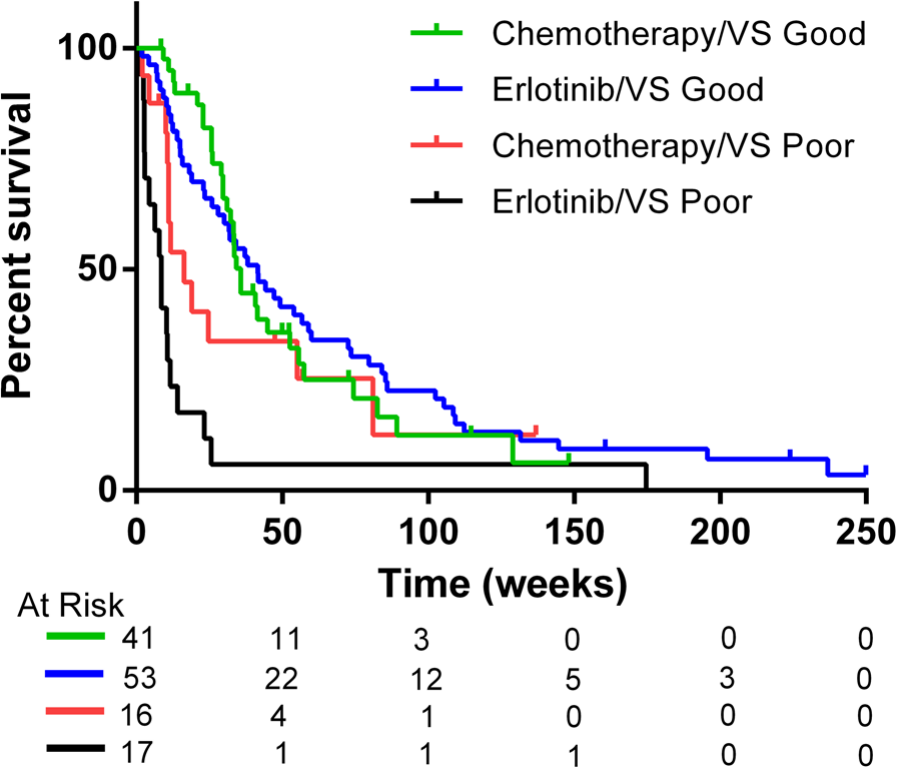
Kaplan-Meier plot of OS by VeriStrat classification and treatment group

The median progression-free survival (PFS) and overall survival (OS) for the entire cohort were 10.7 weeks (95% CI: 8.3–12.6) and 31.7 weeks (95% CI: 25.6–38.1), respectively. No significant difference in OS was detected between treatment groups. However, dramatic differences were detected between the VSG and VSP groups (Fig. 1 and Table 2). Median OS in the erlotinib cohort was 41.6 weeks and 8.6 weeks for VSG and VSP groups, and 35.7 weeks and 16.3 weeks, respectively, within the chemotherapy cohort. A significant interaction between VeriStrat classification and OS was observed when adjusted for baseline patient characteristics (*p* = 0.0035). Gender and smoking (never vs. ever) were also identified as independent predictors of OS (*p* = 0.0262 and 0.0056, respectively). These findings are illustrated via Kaplan-Meier plots as Fig. 1. Similar findings were revealed with our evaluation of PFS, as shown in Additional file 3: Figure S1.

**Table 2:** Analysis of overall survival by VeriStrat classification and treatment

Covariate	Group	Cox PH *p* value	Log Rank *p* value
VeriStrat	Good	0.0002	0.0001
	Poor		
Treatment	Erlotinib	0.6043	0.5985
	Chemotherapy		
Erlotinib	Good	< 0.0001	< 0.0001
	Poor		
Chemotherapy	Good	0.2556	0.2520
	Poor		

### Association of biomarkers with clinical outcome

Circulating levels of 27 biomarkers were found to be significantly associated with OS (Cox PH *p*-value ≤0.05 with FDR < 0.20): of these 16 showed a Cox PH *p*-value < 0.01 and FDR < 0.05. (See Table 3). Nine markers possessed a *p*-value < 0.001, including several biomarkers primarily associated with proinflammatory/ acute phase reactants (CRP, SAA, ferritin, TNFRI, IL-2R_α_, and IL-1RII), The balance of the markers were associated with angiogenesis (thrombospondin-2, PLGF, and angiopoietin-2) or an indirect measure of an acute phase response (e.g. procalcitonin). Very similar findings in terms of biomarkers and processes being represented were obtained when examining PFS, but only 16 biomarkers showed a Cox PH *p*-value ≤0.05. A complete list of these associations is shown in the Supplemental Results section as Additional file 4: Table S3 and Additional file 5: Table S4.

**Table 3:** Significant associations of biomarkers with overall survival

Analyte	Cox PH *p*-value	FDR
thrombospondin-2	< 0.0001	< 0.01
C-reactive protein	< 0.0001	< 0.01
TNF-RI	< 0.0001	< 0.01
PLGF	< 0.0001	< 0.01
angiopoietin-2	0.0001	< 0.01
serum amyloid-A	0.0002	< 0.01
procalcitonin	0.0008	< 0.01
IL-2R_α_	0.0008	< 0.01
IL-1RII	0.0008	< 0.01
suPAR	0.0011	< 0.05
TNFRII	0.0017	< 0.05
TRAIL	0.0020	< 0.05
TPA	0.0025	< 0.05
CYFRA 21-1	0.0026	< 0.05
ferritin	0.0055	< 0.05
sE-selectin	0.0075	< 0.05
sVEGFR1	0.0111	< 0.10
IL-6	0.0140	< 0.10
IGFBP-4	0.0148	< 0.10
osteopontin	0.0198	< 0.10
CA-125	0.0208	< 0.10
IGFBP-3	0.0301	< 0.15
leptin	0.0342	< 0.15
resistin	0.0352	< 0.15
GLP-1	0.0424	< 0.20
prolactin	0.0467	< 0.20
adiponectin	0.0494	< 0.20

### Association of biomarkers with VeriStrat classification

**Fig. 2:**
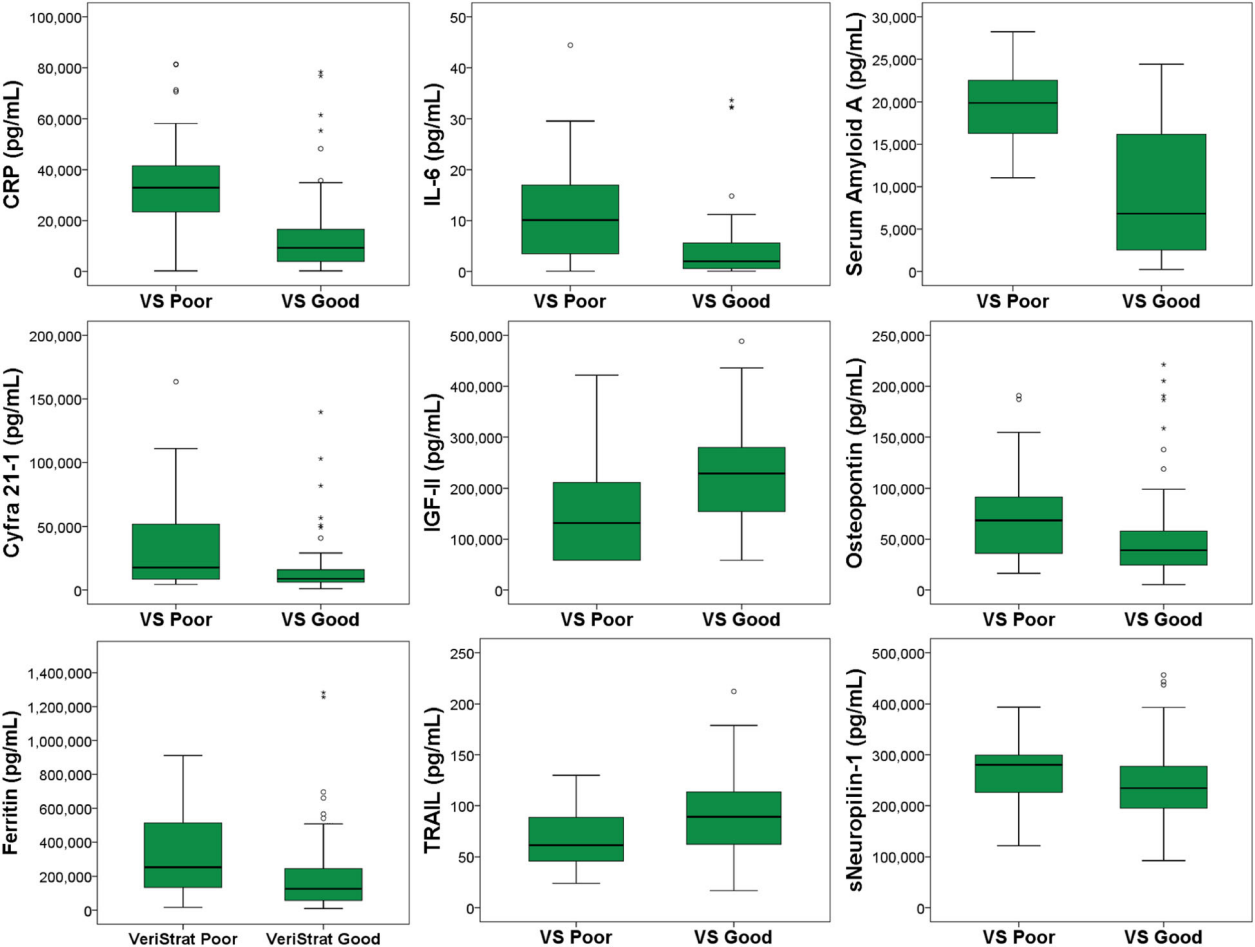
Box and whisker plots of biomarker levels in association with VeriStrat status

A total of 23 significant associations between VS classification and circulating biomarker levels were identified in the present study by a Mann-Whitney Rank Sum test (i.e., *p* ≤ 0.05) (Table 4). These had FDR below 20%. The complete list of associations is included in Additional file 6: Table S5. Biomarkers highly associated with VS classification status (*p* ≤ 0.001) include CRP, IL-6, SAA, CYFRA 21-1, IGF-II, osteopontin, and ferritin. Other biomarkers associated with VS classification were TRAIL, sNeuropilin-1, TPA, resistin, visfatin, IGF-I, sRAGE, IL-2R_α_, thrombospondin-2, BMP-9, procalcitonin, sVEGFR2, IGFBP-5, IL-8, adipsin, and sHER-2. The association of the circulating biomarkers with VS classification possessing a Mann-Whitney *p* < 0.01 are illustrated in Fig. 2 as box and whisker plots. Findings of the associations with *p* ≤ 0.05 are also illustrated as a heatmap with hierarchical clustering, shown as Additional file 7: Figure S2.

**Table 4:** Biomarker association with VeriStrat classification

Analyte	Kruskal-Wallis *p*-value	Mann-Whitney *p*-value	FDR^a^
CRP	<.0001	<.0001	< 0.01
IL-6	<.0001	<.0001	< 0.01
serum amyloid A	<.0001	<.0001	< 0.01
CYFRA 21-1	0.0003	0.0005	< 0.01
IGF-II	0.0003	0.0005	< 0.01
osteopontin	0.0004	0.0006	< 0.01
ferritin	0.0010	0.0013	< 0.05
TRAIL	0.0015	0.0019	< 0.05
sNeuropilin-1	0.0079	0.0090	< 0.10
TPA	0.0101	0.0113	< 0.15
resistin	0.0110	0.0123	< 0.15
visfatin	0.0138	0.0152	< 0.15
IGF-I	0.0151	0.0166	< 0.15
sRAGE	0.0200	0.0218	< 0.15
IL-2Rα	0.0203	0.0221	< 0.15
thrombospondin-2	0.0230	0.0248	< 0.15
BMP-9	0.0242	0.0261	< 0.15
procalcitonin	0.0252	0.0271	< 0.15
sVEGFR2	0.0265	0.0285	< 0.15
IGFBP-5	0.0309	0.0330	< 0.15
IL-8	0.0312	0.0333	< 0.15
adipsin	0.0436	0.0461	< 0.20
sHer-2	0.0472	0.0497	< 0.20

^a^For Mann-Whitney test

limited to those with *p* < 0.05

## Discussion

The predictive and prognostic value of the VeriStrat (VS) test for pretreated advanced NSCLC patients with wild-type EGFR tumors have been extensively studied since the test was first introduced in 2007, as recently reviewed in an editorial by Soo and Adjei [19]. Though prognostic information is certainly useful for counseling NSCLC patients, identifying some of the key (mechanistic) drivers underlying the VeriStrat poor (VSP) classification could open the door for patient selection for novel therapies that might improve outcomes. With the strong prognostic utility of the VS test, the VSG population would likely benefit from most standard of care based therapies, with cautious introduction of agents to improve response. In our study, we have demonstrated that VSG patients benefits from both EGFR TKI as well as single-agent chemotherapy in the EGFR WT population. And while the cohorts evaluated in this study were treated prior to FDA approval of nivolumab for pretreated NSCLC, we are very interested in further evaluating the VS test, and the selected serum biomarkers, to cohorts receiving PD-1/−L1 directed immunotherapy. In this, we anticipate that VS good patients may receive more benefit from immune checkpoint inhibition therapy due to less chronic inflammation and suppression of cytotoxic T cell activity. The current study was developed to investigate potential correlations between specific peptide and protein biomarkers and VS classification. These findings might generate hypotheses regarding mechanisms of tumor progression and novel therapeutic interventions.

Our patient population was unremarkable in terms of clinical characteristics and prevalence of VS status relative to other studies evaluating the predictive and/or prognostic value of VS in pretreated NSCLC patients [3, 6]. The study confirmed the results of the PROSE study [6] which demonstrated the predictive ability of the VS test for differential survival benefit between erlotinib and single agent chemotherapy. Note that although there was no stratified randomization between treatments in this study, the interaction between VS classification and treatment was significant when adjusted for clinical characteristics. The prognostic power of the test for EGFR TKI therapy found in multiple previous studies [1–6] was also confirmed. A majority of the circulating protein biomarkers significantly associated with outcome were proinflammatory/ acute phase reactants. The acute phase response is commonly associated with infection, trauma, inflammatory diseases and cancer [20, 21]. Furthermore, these acute phase reactants accompany both acute and chronic inflammatory states, which are known to promote carcinogenesis and enable cancer characteristics [22]. Not surprisingly, multiple acute phase reactants have been demonstrated to be correlated with poor prognosis in cancer (e.g. CRP, SAA) [23–26]. To the best of our knowledge, the VS test is the only multivariate test capturing the acute phase response with broad applicability for use in clinical practice.

In an attempt to further understand some of the biological processes that may be surveyed by this study, we provide a preliminary account of biomarker mapping to biological processes in the Supplemental Results using the Ingenuity Pathway Analysis Suite and a Gene Set Enrichment (GSE) (Additional file 8: Table S6 and Additional file 9: Table S7, respectively). In these preliminary analyses, ‘fibrotic processes’ was another prominent theme in the biomarkers associating with the VeriStrat status, although this finding may simply reflect the presence of circulating biomarkers of an epithelial-to-mesenchymal transition underlying fibrosis [27]. And while not annotated by these analyses, cancer cachexia emerges as a system-level process highly implicated by these findings; particularly with the theme combining acute phase reactants (e.g. thrombospondin-2, C-reactive protein, serum amyloid A, ferritin), inflammation (e.g. suPAR, IL-6, procalcitonin), adipokines (e.g. leptin, resistin, adiponectin), and metabolic control (e.g. IGFBP-4, IGFBP-3, GLP-1) emerging as a prominent signature [with significance of each listed example provided in Table 3]. Please note these analyses are meant to help promote a mechanistic understanding of the observations from this study and are limited in their scope and should be interpreted with some restraint.

Recently, we described negative associations between increasing neutrophil to lymphocyte ratios (NLR) and declining body weight changes and overall survival in a cohort of advanced NSCLC patients receiving chemotherapy [28]. These observations, together with the known involvement of inflammation and cancer cachexia in advanced NSCLC, suggest that VS could aid in the identification of patients with cachexia and pre-cachexia who are candidates for anti-cachexic agents, such as anamorelin [29, 30]. Inflammatory cytokines [31], inflammatory cells [31, 32], and sarcopenia/cachexia [33] are also implicated in impaired anti-tumor response. Additional study of circulating proteins might identify therapeutic strategies which could positively impact cachexia and anti-tumor immune response.

Finally, some of the themes we describe above also followed through to the associations between VS classifications and circulating biomarker levels, where two prominent hierarchical clusters emerge when illustrated as the heatmap, provided as Additional file 7: Figure S2. Namely, cluster 1 included clearly elevated levels of serum amyloid A, C-reactive protein, ferritin, tissue plasminogen activator, IL-6, and calcitonin in the VSP group, relative to the VSG cases. In cluster 2 the VeriStrat good group was observed to have elevated levels of BMP-9, sRAGE, sVEGFR2, sHER-2, IGF-I, IGF-II, and adipsin, relative to the VSP cases. [Note: the *p* value for the association of each biomarker with VS classification is provided in Table 4 with the complete list provided as Additional file 6: Table S5]. This figure also illustrates nicely the patient-to-patient variations in individual biomarker expression regardless of VS status and stresses the importance of considering multiple analytes in any classification algorithm.

## Conclusions

These findings confirm the prognostic and predictive role of the VS test, as evident by the better outcomes in patients classified as VSG versus VSP in the erlotinib-treated cohort and the differential survival benefit of chemotherapy and erlotinib between VS classifications. In addition, we identified several inflammatory and angiogenic proteins that are associated with VS classification. Though the number of patients in this study is relatively small, further work in this area may elucidate specific, potentially targetable pathways and processes that could improve outcomes for NSCLC patients classified as VSP.

## Additional files

Additional file 1: **Table S1.** Patient Characteristics by VeriStrat status. (DOCX 19 kb)

Additional file 2: **Table S2.** Cohort analysis by VeriStrat classification, PFS, and treatment. (DOCX 17 kb)

Additional file 3: **Figure S1.** Kaplan-Meier plot of PFS by VeriStrat classification and treatment groups. (DOCX 120 kb)

Additional file 4: **Table S3.** Biomarker Association with Overall Survival. (DOCX 21 kb)

Additional file 5: **Table S4.** Biomarker Association with Progression-Free Survival. (DOCX 20 kb)

Additional file 6: **Table S5.** Biomarker Association with VeriStrat Classification. (DOCX 21 kb)

Additional file 7: **Figure S2.** Heatmap of significant associations between biomarkers and VeriStrat status. (DOCX 559 kb)

Additional file 8: **Table S6.** Results from Ingenuity Pathway Analysis Suite Analysis of Biomarker data. (DOCX 18 kb)

Additional file 9: **Table S7.** Gene set enrichment analysis results based on biomarker association with VeriStrat status. (DOCX 18 kb)

## General abbreviations

CGA: the cancer genome atlas
EGFR: epidermal growth factor receptor
EMT: epithelial-mesenchymal transition
NSCLC: Non-Small Cell Lung Cancer
OS: overall survival
PFS: progression free survival
TKI: tyrosine kinase inhibitor

## Biomarker abbreviations

bFGF: basic Fibroblast Growth Factor (aka FGF-2)
CEA: Carcinoembryonic Antigen
EGF: Epidermal Growth Factor
FGF: Fibroblast Growth Factor
GRO: CXCL-1
HB-EGF: Heparin Binding EGF
HCG: Human Chorionic Gonadotropin
HE4: Human Epididymis Protein 4
HGF: Hepatocyte Growth Factor
IGF: Insulin-like Growth Factor
IGFBP: Insulin-like Growth Factor Binding Protein
MIF: Macrophage Migration Inhibitory Factor
MMP: Matrix Metalloproteinase
OPN: Osteopontin
PDGF: Platelet-Derived Growth Factor
PLGF: Placental Growth Factor
PSA: Prostate-specific Antigen
SCF: Stem Cell Factor
sEGFR: soluble Epidermal Growth Factor Receptor
sIL-1RI: soluble Interleukin − 1 Receptor I
sRAGE: soluble Receptor for Advanced Glycation End-products
sTNFRI: soluble Tumor Necrosis Factor Receptor I
sVEGFRI: soluble Vascular-Endothelial Growth Factor Receptor I
TGF-α: Tumor Necrosis Factor-alpha

## References

[1] Taguchi F, Solomon B, Gregorc V, Roder H, Gray R, Kasahara K, Nishio M, Brahmer J, Spreafico A, Ludovini V (2007). Mass spectrometry to classify non-small-cell lung cancer patients for clinical outcome after treatment with epidermal growth factor receptor tyrosine kinase inhibitors: a multicohort cross-institutional study. J Natl Cancer Inst.

[2] Amann JM, Lee JW, Roder H, Brahmer J, Gonzalez A, Schiller JH, Carbone DP (2010). Genetic and proteomic features associated with survival after treatment with erlotinib in first-line therapy of non-small cell lung cancer in eastern cooperative oncology group 3503. J Thorac Oncol.

[3] Carbone DP, Ding K, Roder H, Grigorieva J, Roder J, Tsao MS, Seymour L, Shepherd FA (2012). Prognostic and predictive role of the VeriStrat plasma test in patients with advanced non-small-cell lung cancer treated with erlotinib or placebo in the NCIC clinical trials group BR.21 trial. J Thorac Oncol.

[4] Stinchcombe TE (2016). The use of EGFR tyrosine kinase inhibitors in EGFR wild-type non-small-cell lung Cancer. Curr Treat Options in Oncol.

[5] Wakelee H, Goldman JW, Gadgeel S, Camidge DR, Reckamp KL, Ou SI, Yu HA, Solomon B, Liu SV, Perol M (2016). PS01.66: biomarker stratification of outcomes of third-generation EGFR TKI therapy in patients with previously-treated advanced NSCLC: Topic: Medical Oncology. J Thorac Oncol.

[6] Gregorc V, Novello S, Lazzari C, Barni S, Aieta M, Mencoboni M, Grossi F, De Pas T, de Marinis F, Bearz A (2014). Predictive value of a proteomic signature in patients with non-small-cell lung cancer treated with second-line erlotinib or chemotherapy (PROSE): a biomarker-stratified, randomised phase 3 trial. Lancet Oncol.

[7] Akerley W, Boucher K, Rich N, Egbert L, Harker G, Bylund J, Van Duren T, Reddy C (2013). A phase II study of bevacizumab and erlotinib as initial treatment for metastatic non-squamous, non-small cell lung cancer with serum proteomic evaluation. Lung Cancer.

[8] Carbone DP, Salmon JS, Billheimer D, Chen H, Sandler A, Roder H, Roder J, Tsypin M, Herbst RS, Tsao AS (2010). VeriStrat classifier for survival and time to progression in non-small cell lung cancer (NSCLC) patients treated with erlotinib and bevacizumab. Lung Cancer.

[9] Gautschi O, Dingemans AM, Crowe S, Peters S, Roder H, Grigorieva J, Roder J, Zappa F, Pless M, Brutsche M (2013). VeriStrat(R) has a prognostic value for patients with advanced non-small cell lung cancer treated with erlotinib and bevacizumab in the first line: pooled analysis of SAKK19/05 and NTR528. Lung Cancer.

[10] Kuiper JL, Lind JS, Groen HJ, Roder J, Grigorieva J, Roder H, Dingemans AM, Smit EF (2012). VeriStrat((R)) has prognostic value in advanced stage NSCLC patients treated with erlotinib and sorafenib. Br J Cancer.

[11] Molina-Pinelo S, Pastor MD, Paz-Ares L (2014). VeriStrat: a prognostic and/or predictive biomarker for advanced lung cancer patients?. Expert Rev Respir Med.

[12] Grossi F, Rijavec E, Genova C, Barletta G, Biello F, Maggioni C, Burrafato G, Sini C, Dal Bello MG, Meyer K (2017). Serum proteomic test in advanced non-squamous non-small cell lung cancer treated in first line with standard chemotherapy. Br J Cancer.

[13] Vansteenkiste J, Paz-Ares L, Eisen T, Heigener D, Eberhardt R, Thomas M, Zhou C, Santoro A, Lathia C, Roder H (2012). A plasma proteomic signature predicts outcomes in a Phase 3 study of gemcitabine (G)+cisplatin (C)±sorafenib in first line Stage IIIB or IV NSCLC. Ann Onc.

[14] Grossi F, Rijavec E, Biella F, Barletta G, Maggioni C, Genova C, Giovanna Dal Bello M, Rossi G, Distefano R, Roder J (2017). P3.02c-074 Evaluation of a Pretreatment Serum Tests for Nivolumab Benefit in Patients with Non-Small Cell Lung Cancer. J Thorac Oncol.

[15] Milan E, Lazzari C, Anand S, Floriani I, Torri V, Sorlini C, Gregorc V, Bachi A. SAA1 is over-expressed in plasma of non small cell lung cancer patients with poor outcome after treatment with epidermal growth factor receptor tyrosine-kinase inhibitors. J Proteomics. 2012;76 Spec No.:91–101 10.1016/j.jprot.2012.06.022 22771314

[16] Fidler MJ, Frankenberger C, Seto R, Lobato GC, Fhied CL, Sayidine S, Basu S, Pool M, Karmali R, Batus M, Lie WR, Hayes D, Mistry J, Bonomi P, Borgia JA. Differential expression of circulating biomarkers of tumor phenotype and outcomes in previously treated non-small cell lung cancer patients receiving erlotinib vs. cytotoxic chemotherapy. Oncotarget. 2017;8(35):58108–21. 10.18632/oncotarget.17510 PMC5601637 28938541

[17] Buckingham LE, Coon JS, Morrison LE, Jacobson KK, Jewell SS, Kaiser KA, Mauer AM, Muzzafar T, Polowy C, Basu S (2007). The prognostic value of chromosome 7 polysomy in non-small cell lung cancer patients treated with gefitinib. J Thorac Oncol.

[18] Benjamini Y, Hochberg Y (1995). Controlling the false discovery rate: a practical and powerful approach to multiple testing. J R Statist Soc B.

[19] Soo RA, Adjei AA (2017). Predicting clinical outcomes using proteomics in non-small cell lung Cancer-the past, present, and future. J Thorac Oncol.

[20] Gabay C, Kushner I (1999). Acute-phase proteins and other systemic responses to inflammation. N Engl J Med.

[21] Kushner I (1982). The phenomenon of the acute phase response. Ann N Y Acad Sci.

[22] Hanahan D, Weinberg RA (2011). Hallmarks of cancer: the next generation. Cell.

[23] Biran H, Friedman N, Neumann L, Pras M, Shainkin-Kestenbaum R (1986). Serum amyloid a (SAA) variations in patients with cancer: correlation with disease activity, stage, primary site, and prognosis. J Clin Pathol.

[24] Cho WC, Yip TT, Cheng WW, Au JS (2010). Serum amyloid a is elevated in the serum of lung cancer patients with poor prognosis. Br J Cancer.

[25] Findeisen P, Zapatka M, Peccerella T, Matzk H, Neumaier M, Schadendorf D, Ugurel S (2009). Serum amyloid a as a prognostic marker in melanoma identified by proteomic profiling. J Clin Oncol.

[26] Heikkila K, Ebrahim S, Lawlor DA (2007). A systematic review of the association between circulating concentrations of C reactive protein and cancer. J Epidemiol Community Health.

[27] Kalluri R, Weinberg RA (2009). The basics of epithelial-mesenchymal transition. J Clin Invest.

[28] Derman BA, Macklis JN, Azeem MS, Sayidine S, Basu S, Batus M, Esmail F, Borgia JA, Bonomi P, Fidler MJ (2017). Relationships between longitudinal neutrophil to lymphocyte ratios, body weight changes, and overall survival in patients with non-small cell lung cancer. BMC Cancer.

[29] Bai Y, Hu Y, Zhao Y, Yu X, Xu J, Hua Z, Zhao Z (2017). Anamorelin for cancer anorexia-cachexia syndrome: a systematic review and meta-analysis. Support Care Cancer.

[30] Temel JS, Abernethy AP, Currow DC, Friend J, Duus EM, Yan Y, Fearon KC (2016). Anamorelin in patients with non-small-cell lung cancer and cachexia (ROMANA 1 and ROMANA 2): results from two randomised, double-blind, phase 3 trials. Lancet Oncol.

[31] Chen DS, Mellman I (2013). Oncology meets immunology: the cancer-immunity cycle. Immunity.

[32] Coffelt SB, Wellenstein MD, de Visser KE (2016). Neutrophils in cancer: neutral no more. Nat Rev Cancer.

[33] Dercle L, Ammari S, Champiat S, Massard C, Ferte C, Taihi L, Seban RD, Aspeslagh S, Mahjoubi L, Kamsu-Kom N (2016). Rapid and objective CT scan prognostic scoring identifies metastatic patients with long-term clinical benefit on anti-PD-1/−L1 therapy. Eur J Cancer.

