# Supplementary material for: The serum-based VeriStrat® test is associated with proinflammatory reactants and clinical outcome in non-small cell lung cancer patients
Source: BMC Cancer. 2018 Mar 20;18:310. doi: 10.1186/s12885-018-4193-0 (PMC5861613; doi:10.1186/s12885-018-4193-0)
Supplement: Supplementary file 1 — Table S1. Patient Characteristics by VeriStrat status. (DOCX 19 kb) [file 12885_2018_4193_MOESM1_ESM.docx]

**Table SI Patient Characteristics by VeriStrat status**

|  | Good  (n = 94) | Poor  (n = 33) | p value |
| --- | --- | --- | --- |
|  |  |  |  |
| **Age** |  |  |  |
| Mean (SD) | 63.8 (9.2) | 64.8 (9.8) | 0.6125 |
| Median (Range) | 64.5 (40.9-83.7) | 66.5 (41.2-88.2) | 0.6908 |
| **Gender, n (%)** |  |  | 0.1044 |
| Female | 54 (57.4) | 13 (39.4) |  |
| Male | 40 (42.6) | 20 (60.6) |  |
| **Race** |  |  | 0.7213 |
| White | 70 (74.5) | 22 (66.7) |  |
| Black | 21 (22.3) | 10 (30.3) |  |
| Asian/Pacific Islander | 2 (2.1) | 1 (3.0) |  |
| Asian | 1 (1.1) | 0 (0) |  |
| **Histology, n (%)** |  |  | 0.2424 |
| Adenocarcinoma | 63 (67.0) | 17 (51.5) |  |
| Adenosquamous | 2 (2.1) | 0 (0) |  |
| Bronchioalveolar | 1 (1.1) | 0 (0) |  |
| Bronchogenic carcinoma | 1 (1.1) | 0 (0) |  |
| Carcinoma | 9 (9.6) | 6 (18.2) |  |
| Large Cell | 0 (0) | 1 (3.0) |  |
| NSCLC | 1 (1.1) | 2 (6.1) |  |
| Neuroendocrine | 1 (1.1) | 0 (0) |  |
| Squamous | 16 (17.0) | 7 (21.2) |  |
| **Smoking Status, n (%)** |  |  | >0.9999 |
| Yes | 81 (86.2) | 29 (87.9) |  |
| No | 12 (12.8) | 4 (12.1) |  |
| Missing | 1 (1.1) | 0 (0) |  |
| **Performance Status, n (%)** |  |  | 0.0807 |
| 0 | 21 (22.3) | 7 (21.2) |  |
| 0.5 | 0 (0) | 1 (3.0) |  |
| 1 | 62 (70.0) | 18 (54.5) |  |
| 1.5 | 1 (1.1) | 1 (3.0) |  |
| 2 | 10 (10.7) | 4 (12.1) |  |
| 3 | 0 (0) | 2 (6.1) |  |
| **Grade n (%)** |  |  | 0.7246 |
| Moderately | 14 (14.9) | 2 (6.1) |  |
| Moderately/Poorly | 4 (4.3) | 1 (3.0) |  |
| Nos | 39 (41.5) | 13 (39.4) |  |
| Poorly | 31 (33.0) | 15 (45.5) |  |
| Well | 5 (5.3) | 2 (6.1) |  |
| Well/Moderately | 1 (1.1) | 0 (0) |  |
|  |  |  |  |
|  |  |  |  |
|  |  |  |  |
