# Supplementary material for: The serum-based VeriStrat® test is associated with proinflammatory reactants and clinical outcome in non-small cell lung cancer patients
Source: BMC Cancer. 2018 Mar 20;18:310. doi: 10.1186/s12885-018-4193-0 (PMC5861613; doi:10.1186/s12885-018-4193-0)
Supplement: Supplementary file 2 — Table S2. Cohort analysis by VeriStrat classification, PFS, and treatment. (DOCX 17 kb) [file 12885_2018_4193_MOESM2_ESM.docx]

**Table S2 Cohort analysis by VeriStrat classification, PFS, and treatment**

| **Covariate** | **Group** | **Median OS in Weeks (95% CI)** | **HR (95% CI)** | **Cox PH**  **p value** | **log rank p value** |
| --- | --- | --- | --- | --- | --- |
| VeriStrat | Good | 12.7 (10.3-16.3) | 0.45 (0.30-0.68) | 0.0001 | <.0001 |
|  | Poor | 6.1 (4.0-8.3) |  |  |  |
| Treatment | Erlotinib | 8.1 (7.1-11.9) | 0.80 (0.56-1.15) | 0.2267 | 0.2287 |
|  | Chemotherapy | 12.4 (9.9-18.6) |  |  |  |
| Erlotinib | Good | 11.3 (8.0-15.0) | 0.61 (0.34-1.09 | 0.0963 | 0.0952 |
|  | Poor | 5.1 (2.7-8.0) |  |  |  |
| Chemotherapy | Good | 15.1 (11.7-22.4) | 0.32 (0.18-0.58) | 0.0001 | <0.0001 |
|  | Poor | 8.7 (4.0-12.6) |  |  |  |
