# Supplementary material for: The serum-based VeriStrat® test is associated with proinflammatory reactants and clinical outcome in non-small cell lung cancer patients
Source: BMC Cancer. 2018 Mar 20;18:310. doi: 10.1186/s12885-018-4193-0 (PMC5861613; doi:10.1186/s12885-018-4193-0)
Supplement: Supplementary file 3 — Figure S1. Kaplan-Meier plot of PFS by VeriStrat classification and treatment groups. (DOCX 120 kb) [file 12885_2018_4193_MOESM3_ESM.docx]

**Figure S1** Kaplan-Meier plot of PFS by VeriStrat classification and treatment groups, as indicated.

**

**
