# Supplementary material for: The serum-based VeriStrat® test is associated with proinflammatory reactants and clinical outcome in non-small cell lung cancer patients
Source: BMC Cancer. 2018 Mar 20;18:310. doi: 10.1186/s12885-018-4193-0 (PMC5861613; doi:10.1186/s12885-018-4193-0)
Supplement: Supplementary file 4 — Table S3. Biomarker Association with Overall Survival. (DOCX 21 kb) [file 12885_2018_4193_MOESM4_ESM.docx]

**Table S3 Biomarker Association with Overall Survival.** Complete set of findings with all biomarkers meeting the study criteria (as defined in Methods) represented.

| **Analyte** | **Cox PH p-value** | **FDR** |
| --- | --- | --- |
| C-reactive protein | <0.0001 | <0.01 |
| PLGF | <0.0001 | <0.01 |
| Thrombospondin-2 | <0.0001 | <0.01 |
| TNF-RI | <0.0001 | <0.01 |
| angiopoietin-2 | 0.0001 | <0.01 |
| serum amyloid-A | 0.0002 | <0.01 |
| IL-1RII | 0.0008 | <0.01 |
| IL-2R_α_ | 0.0008 | <0.01 |
| procalcitonin | 0.0008 | <0.01 |
| suPAR | 0.0011 | <0.05 |
| TNFRII | 0.0017 | <0.05 |
| TRAIL | 0.0020 | <0.05 |
| TPA | 0.0025 | <0.05 |
| CYFRA 21-1 | 0.0026 | <0.05 |
| ferritin | 0.0055 | <0.05 |
| sE-Selectin | 0.0075 | <0.05 |
| sVEGFR1 | 0.0111 | <0.10 |
| IL-6 | 0.0140 | <0.10 |
| IGFBP-4 | 0.0148 | <0.10 |
| osteopontin | 0.0198 | <0.10 |
| CA-125 | 0.0208 | <0.10 |
| IGFBP-3 | 0.0301 | <0.15 |
| leptin | 0.0342 | <0.15 |
| resistin | 0.0352 | <0.15 |
| GLP-1 | 0.0424 | <0.20 |
| prolactin | 0.0467 | <0.20 |
| adiponectin | 0.0494 | <0.20 |
| HE4 | 0.0540 | <0.20 |
| ghrelin | 0.0611 | <0.20 |
| tenascin-C | 0.0706 | <0.25 |
| CA15-3 | 0.0833 | <0.25 |
| IGF-II | 0.0943 | <0.30 |
| sNeuropilin-1 | 0.0988 | <0.30 |
| CA-199 | 0.1072 | <0.30 |
| glucagon | 0.1335 | <0.40 |
| CEA | 0.1340 | <0.40 |
| visfatin | 0.1430 | <0.40 |
| sFasL | 0.1506 | <0.40 |
| IL-4R | 0.1807 | <0.50 |
| sAXL | 0.1833 | <0.50 |
| IGF-I | 0.1935 | <0.50 |
| IGFBP-1 | 0.2021 | <0.50 |
| C-peptide | 0.2098 | <0.50 |
| serum Amyloid P | 0.2128 | <0.50 |
| sTie-2 | 0.2169 | <0.50 |
| G-CSF | 0.2268 | <0.50 |
| IL-1RI | 0.2300 | <0.50 |
| BMP-9 | 0.2484 | <0.50 |
| VEGF-D | 0.2709 | <0.50 |
| sHGFR/cMet | 0.2730 | <0.50 |
| sHer-2 | 0.2742 | <0.50 |
| IL-8 | 0.2852 | <0.60 |
| gp130 | 0.2919 | <0.60 |
| VEGF-A | 0.3207 | <0.60 |
| fibrinogen | 0.3238 | <0.60 |
| FGF-2 | 0.3615 | <0.60 |
| IGFBP-2 | 0.3816 | <0.70 |
| Sc-kit/SCFR | 0.3990 | <0.70 |
| angiostatin | 0.4614 | <0.80 |
| follistatin | 0.4883 | <0.80 |
| adipsin | 0.5102 | <0.80 |
| TGF-α | 0.5103 | <0.80 |
| CD30 | 0.5186 | <0.80 |
| sRAGE | 0.5295 | <0.80 |
| IGFBP-7 | 0.5745 | <0.90 |
| sVEGFR3 | 0.5948 | <0.90 |
| alpha-fetoprotein | 0.6029 | <0.90 |
| IGFBP-5 | 0.6043 | <0.90 |
| sPECAM-1 | 0.6105 | <0.90 |
| PDGF-AB/BB | 0.6227 | <0.90 |
| MIF | 0.6523 | <0.90 |
| EGF | 0.6697 | <0.90 |
| sHer-3 | 0.6890 | <0.90 |
| IGFBP-6 | 0.7339 | <1.00 |
| PSA (total) | 0.7472 | <1.00 |
| endoglin | 0.7659 | <1.00 |
| GIP | 0.7789 | <1.00 |
| IL-6R | 0.7876 | <1.00 |
| insulin | 0.7929 | <1.00 |
| endothelin1 | 0.8392 | <1.00 |
| sFas | 0.8614 | <1.00 |
| SCF | 0.8716 | <1.00 |
| VEGF-C | 0.8926 | <1.00 |
| beta-HCG | 0.8932 | <1.00 |
| HB-EGF | 0.8975 | <1.00 |
| sVEGFR2 | 0.8981 | <1.00 |
| FGF-1 | 0.8993 | <1.00 |
| sIL-6Rα | 0.9121 | <1.00 |
| TNF-α | 0.9160 | <1.00 |
| alpha2-macroglobulin | 0.9208 | <1.00 |
| HGF | 0.9739 | <1.00 |
| sEGFR | 0.9973 | <1.00 |
