# Supplementary material for: The serum-based VeriStrat® test is associated with proinflammatory reactants and clinical outcome in non-small cell lung cancer patients
Source: BMC Cancer. 2018 Mar 20;18:310. doi: 10.1186/s12885-018-4193-0 (PMC5861613; doi:10.1186/s12885-018-4193-0)
Supplement: Supplementary file 5 — Table S4. Biomarker Association with Progression-Free Survival. (DOCX 20 kb) [file 12885_2018_4193_MOESM5_ESM.docx]

**Table S4 Biomarker Association with Progression-Free Survival.** Complete set of findings with all biomarkers meeting the study criteria (as defined in Methods) represented.

| **Analyte** | **Cox PH p-value** | **FDR** |
| --- | --- | --- |
| thrombospondin-2 | <0.0001 | <0.01 |
| CRP | 0.0001 | <0.01 |
| PLGF | 0.0001 | <0.01 |
| SAA | 0.0003 | <0.01 |
| TRAIL | 0.0003 | <0.01 |
| leptin | 0.0030 | <0.05 |
| angiopoietin-2 | 0.0035 | <0.05 |
| IGFBP-3 | 0.0101 | <0.15 |
| procalcitonin | 0.0115 | <0.15 |
| IL-1RII | 0.0194 | <0.20 |
| sE-selectin | 0.0201 | <0.20 |
| tenascin-C | 0.0239 | <0.20 |
| TPA | 0.0321 | <0.25 |
| TNFRI | 0.0330 | <0.25 |
| beta-HCG | 0.0388 | <0.25 |
| IL-1RI | 0.0499 | <0.30 |
| sNeuropilin-1 | 0.0635 | <0.40 |
| IL-8 | 0.0675 | <0.40 |
| TNFRII | 0.0769 | <0.40 |
| sFasL | 0.0879 | <0.40 |
| sHGFR/cMet | 0.0888 | <0.40 |
| sVEGFR1 | 0.0934 | <0.40 |
| G-CSF | 0.0961 | <0.40 |
| IGFBP-6 | 0.1264 | <0.50 |
| gp130 | 0.1374 | <0.60 |
| sHer-2 | 0.1872 | <0.70 |
| suPAR | 0.1895 | <0.70 |
| IL-6 | 0.1920 | <0.70 |
| sAXL | 0.2047 | <0.70 |
| CD30 | 0.2229 | <0.70 |
| ferritin | 0.2307 | <0.70 |
| CA-125 | 0.290 | <0.80 |
| sRAGE | 0.2917 | <0.80 |
| VEGF-A | 0.2992 | <0.80 |
| insulin | 0.3102 | <0.80 |
| IGFBP-4 | 0.3130 | <0.80 |
| adiponectin | 0.3404 | <0.80 |
| IL-2R_α_ | 0.3456 | <0.80 |
| osteopontin | 0.3472 | <0.80 |
| SCF | 0.3486 | <0.80 |
| CYFRA 21-1 | 0.3580 | <0.80 |
| FGF-2 | 0.3775 | <0.80 |
| TNF-α | 0.3856 | <0.80 |
| MIF | 0.3866 | <0.80 |
| PDGF-AB/BB | 0.3978 | <0.80 |
| Sc-kit/SCFR | 0.4049 | <0.80 |
| GIP | 0.4069 | <0.80 |
| VEGF-D | 0.4081 | <0.80 |
| alpha-fetoprotein | 0.4183 | <0.80 |
| angiostatin | 0.4210 | <0.80 |
| IGFBP-1 | 0.4510 | <0.80 |
| IGF-II | 0.4637 | <0.80 |
| FGF-1 | 0.4739 | <0.80 |
| sEGFR | 0.4761 | <0.80 |
| alpha2-macroglobulin | 0.4936 | <0.80 |
| BMP-9 | 0.4986 | <0.80 |
| adipsin | 0.5059 | <0.80 |
| sTie-2 | 0.5068 | <0.80 |
| IGF-I | 0.5140 | <0.80 |
| ghrelin | 0.5233 | <0.80 |
| sVEGFR2 | 0.5252 | <0.80 |
| sHer3 | 0.5428 | <0.90 |
| fibrinogen | 0.5481 | <0.90 |
| HE4 | 0.5913 | <0.90 |
| IGFBP-7 | 0.5966 | <0.90 |
| prolactin | 0.5977 | <0.90 |
| TGF-α | 0.6067 | <0.90 |
| HB-EGF | 0.6123 | <0.90 |
| IGFBP-5 | 0.6249 | <0.90 |
| CA 19-9 | 0.6401 | <0.90 |
| sVEGFR3 | 0.6721 | <0.90 |
| VEGF-C | 0.6866 | <0.90 |
| sIL-6Rα | 0.6951 | <0.90 |
| GLP-1 | 0.6969 | <0.90 |
| visfatin | 0.7139 | <0.90 |
| sPECAM-1 | 0.7492 | <0.90 |
| HGF | 0.7499 | <0.90 |
| endothelin-1 | 0.7514 | <0.90 |
| C-peptide | 0.7593 | <0.90 |
| PSA (total) | 0.7939 | <1.00 |
| IL-6R | 0.8095 | <1.00 |
| CA15-3 | 0.8196 | <1.00 |
| serum amyloid P | 0.8243 | <1.00 |
| sFas | 0.8797 | <1.00 |
| IL-4R | 0.8806 | <1.00 |
| IGFBP-2 | 0.9116 | <1.00 |
| CEA | 0.9162 | <1.00 |
| follistatin | 0.9195 | <1.00 |
| EGF | 0.9350 | <1.00 |
| resistin | 0.9377 | <1.00 |
| endoglin | 0.9385 | <1.00 |
| glucagon | 0.955 | <1.00 |
