# Supplementary material for: The serum-based VeriStrat® test is associated with proinflammatory reactants and clinical outcome in non-small cell lung cancer patients
Source: BMC Cancer. 2018 Mar 20;18:310. doi: 10.1186/s12885-018-4193-0 (PMC5861613; doi:10.1186/s12885-018-4193-0)
Supplement: Supplementary file 6 — Table S5. Biomarker Association with VeriStrat Classification. (DOCX 21 kb) [file 12885_2018_4193_MOESM6_ESM.docx]

**Table S5 Biomarker Association with VeriStrat Classification.** Complete set of findings with all biomarkers meeting the study criteria (as defined in Methods) represented.

| **Analyte** | **Kruskal-Wallis** | **Mann-Whitney** | **FDR for Mann-Whitney** |
| --- | --- | --- | --- |
|  | **p-value** | **p-value** |  |
| CRP | <.0001 | <.0001 | <0.01 |
| IL-6 | <.0001 | <.0001 | <0.01 |
| serum amyloid A | <.0001 | <.0001 | <0.01 |
| CYFRA 21-1 | 0.0003 | 0.0005 | <0.01 |
| IGF-II | 0.0003 | 0.0005 | <0.01 |
| osteopontin | 0.0004 | 0.0006 | <0.01 |
| ferritin | 0.001 | 0.0013 | <0.05 |
| TRAIL | 0.0015 | 0.0019 | <0.05 |
| sNeuropilin-1 | 0.0079 | 0.009 | <0.10 |
| TPA | 0.0101 | 0.0113 | <0.15 |
| resistin | 0.011 | 0.0123 | <0.15 |
| visfatin | 0.0138 | 0.0152 | <0.15 |
| IGF-I | 0.0151 | 0.0166 | <0.15 |
| sRAGE | 0.02 | 0.0218 | <0.15 |
| IL-2Rα | 0.0203 | 0.0221 | <0.15 |
| thrombospondin-2 | 0.023 | 0.0248 | <0.15 |
| BMP-9 | 0.0242 | 0.0261 | <0.15 |
| procalcitonin | 0.0252 | 0.0271 | <0.15 |
| sVEGFR2 | 0.0265 | 0.0285 | <0.15 |
| IGFBP-5 | 0.0309 | 0.0330 | <0.15 |
| IL-8 | 0.0312 | 0.0333 | <0.15 |
| adipsin | 0.0436 | 0.0461 | <0.20 |
| sHer-2 | 0.0472 | 0.0497 | <0.20 |
| α2-macroglobulin | 0.0516 | 0.0542 | <0.25 |
| PSA (total) | 0.0526 | 0.0552 | <0.25 |
| sc-kit/SCFR | 0.0655 | 0.0683 | <0.25 |
| TNFRI | 0.0655 | 0.0683 | <0.25 |
| sEGFR | 0.0731 | 0.0759 | <0.25 |
| sFasL | 0.0753 | 0.0781 | <0.25 |
| IGFBP-7 | 0.0863 | 0.0893 | <0.30 |
| fibrinogen | 0.0969 | 0.0999 | <0.30 |
| IGFBP-6 | 0.098 | 0.1010 | <0.30 |
| angiopoietin-2 | 0.1025 | 0.1056 | <0.30 |
| leptin | 0.106 | 0.1091 | <0.30 |
| IGFBP-1 | 0.1125 | 0.1157 | <0.40 |
| suPAR | 0.1198 | 0.1229 | <0.40 |
| VEGF-C | 0.1251 | 0.1283 | <0.40 |
| HGF | 0.1429 | 0.1461 | <0.40 |
| tenascin-C | 0.171 | 0.1743 | <0.50 |
| C-peptide | 0.1798 | 0.1831 | <0.50 |
| Serum Amyloid P | 0.1964 | 0.1997 | <0.50 |
| TGF-α | 0.2174 | 0.2207 | <0.50 |
| CEA | 0.2286 | 0.2319 | <0.50 |
| sE-selectin | 0.2286 | 0.2319 | <0.50 |
| CA15-3 | 0.2551 | 0.2584 | <0.60 |
| α-fetoprotein | 0.263 | 0.2663 | <0.60 |
| CD30 | 0.2644 | 0.2677 | <0.60 |
| MIF | 0.2715 | 0.2748 | <0.60 |
| PLGF | 0.2897 | 0.2930 | <0.60 |
| IL-6R | 0.2912 | 0.2945 | <0.60 |
| TNFRII | 0.317 | 0.3203 | <0.60 |
| CA-125 | 0.3197 | 0.3230 | <0.60 |
| FGF-2 | 0.3196 | 0.3230 | <0.60 |
| sAXL | 0.3278 | 0.3310 | <0.60 |
| sPECAM-1 | 0.3305 | 0.3338 | <0.60 |
| HB-EGF | 0.3333 | 0.3365 | <0.60 |
| endothelin-1 | 0.3343 | 0.3376 | <0.60 |
| IL-1RII | 0.4065 | 0.4096 | <0.70 |
| sVEGFR3 | 0.4174 | 0.4205 | <0.70 |
| IL-1RI | 0.4302 | 0.4333 | <0.70 |
| EGF | 0.4503 | 0.4534 | <0.70 |
| prolactin | 0.4514 | 0.4544 | <0.70 |
| angiostatin | 0.4525 | 0.4557 | <0.70 |
| VEGF-A | 0.4664 | 0.4698 | <0.70 |
| adiponectin | 0.468 | 0.4711 | <0.70 |
| beta-HCG | 0.4742 | 0.4773 | <0.70 |
| sHer-3 | 0.4767 | 0.4797 | <0.70 |
| insulin | 0.4851 | 0.4881 | <0.70 |
| sTie-2 | 0.5345 | 0.5374 | <0.80 |
| IGFBP-2 | 0.5399 | 0.5428 | <0.80 |
| HE4 | 0.5428 | 0.5459 | <0.80 |
| follistatin | 0.5454 | 0.5483 | <0.80 |
| sVEGFR1 | 0.5619 | 0.5648 | <0.80 |
| GIP | 0.5638 | 0.5667 | <0.80 |
| FGF-1 | 0.5896 | 0.5939 | <0.80 |
| VEGF-D | 0.6344 | 0.6372 | <0.80 |
| PDGF-AB/BB | 0.6482 | 0.6509 | <0.80 |
| CA19-9 | 0.6501 | 0.6529 | <0.80 |
| IL-4R | 0.6581 | 0.6608 | <0.80 |
| GLP-1 | 0.6841 | 0.6868 | <0.80 |
| glucagon | 0.7065 | 0.7091 | <0.90 |
| endoglin | 0.7188 | 0.7214 | <0.90 |
| ghrelin | 0.7415 | 0.7441 | <0.90 |
| sHGFR/cMet | 0.775 | 0.7775 | <0.90 |
| gp130 | 0.7792 | 0.7818 | <0.90 |
| IGFBP-3 | 0.7792 | 0.7818 | <0.90 |
| IGFBP-4 | 0.8687 | 0.8711 | <1.00 |
| sIL-6Rα | 0.9168 | 0.9192 | <1.00 |
| sFas | 0.9212 | 0.9235 | <1.00 |
| TNF-α | 0.9452 | 0.9475 | <1.00 |
| G-CSF | 0.9846 | 0.9869 | <1.00 |
| SCF | 0.9934 | 0.9956 | <1.00 |
