# Supplementary material for: The serum-based VeriStrat® test is associated with proinflammatory reactants and clinical outcome in non-small cell lung cancer patients
Source: BMC Cancer. 2018 Mar 20;18:310. doi: 10.1186/s12885-018-4193-0 (PMC5861613; doi:10.1186/s12885-018-4193-0)

**Figure S2 Heatmap of significant associations between biomarkers and VeriStrat.** Illustration of heatmap of associations (p≤0.05) between biomarkers and VeriStrat with hierarchical clustering indicated. Biomarker levels have been standardized (z-scores).


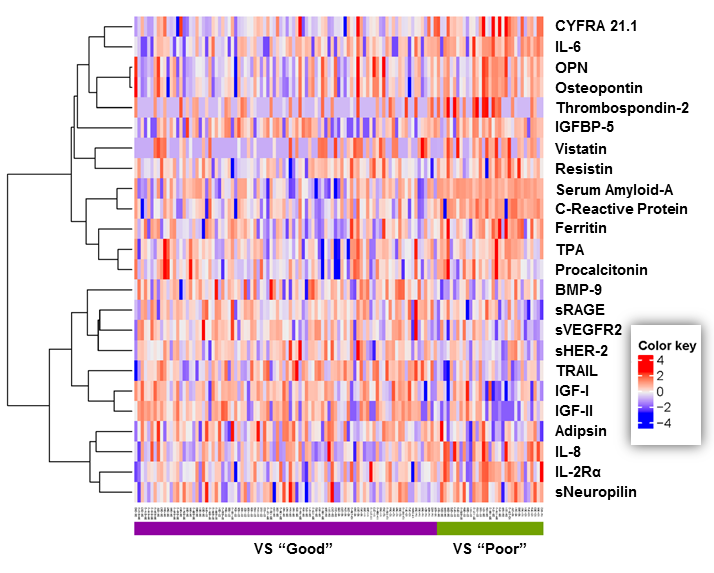

Supplement: Supplementary file 7 — Figure S2. Heatmap of significant associations between biomarkers and VeriStrat status. (DOCX 559 kb) [file 12885_2018_4193_MOESM7_ESM.docx]
