# Supplementary material for: The serum-based VeriStrat® test is associated with proinflammatory reactants and clinical outcome in non-small cell lung cancer patients
Source: BMC Cancer. 2018 Mar 20;18:310. doi: 10.1186/s12885-018-4193-0 (PMC5861613; doi:10.1186/s12885-018-4193-0)
Supplement: Supplementary file 8 — Table S6. Results from Ingenuity Pathway Analysis Suite Analysis of Biomarker data. (DOCX 18 kb) [file 12885_2018_4193_MOESM8_ESM.docx]

**Table S6 Results from Ingenuity Pathway Analysis Suite Analysis of Biomarker data.** The IPA Suite (Spring 2017 release - build 439932M; Qiagen Inc., Germantown, MD) was used to identify the ‘Top Canonical Pathways’ for biomarkers found to be significant and listed in **Table III.** All biomarkers were considered and the software set to consider only molecules and/or relationships where species = Human AND confidence = High (Predicted) or Experimentally Observed. Only pathways with p≤0.001 are shown and biomarker data was uncorrected for multiple comparisons.

| **Ingenuity Canonical Pathways** | **-log**  **(p-value)** | **Ratio** | **Molecules (UniProt IDs)** |
| --- | --- | --- | --- |
| Hepatic Fibrosis / Hepatic Stellate Cell Activation | 11.3 | 0.0497 | CXCL8,IGF2,IGF1,TNFRSF1A,IGFBP5,IL6,A2M,  FASLG, EGFR |
| Acute Phase Response Signaling | 8.25 | 0.0417 | FTL,TNFRSF1A,SAA1,CRP,IL6,FGA,A2M |
| IL-6 Signaling | 5.85 | 0.0394 | CXCL8,TNFRSF1A,CRP,IL6,A2M |
| HMGB1 Signaling | 5.79 | 0.0382 | CXCL8,AGER,TNFRSF1A,IL6,PLAT |
| Coagulation System | 4.67 | 0.0857 | FGA,A2M,PLAT |
| Role of IL-17F in Allergic Inflammatory Airway Diseases | 4.46 | 0.0732 | CXCL8,IGF1,IL6 |
| LXR/RXR Activation | 4.46 | 0.0331 | TNFRSF1A,SAA1,IL6,FGA |
| T Helper Cell Differentiation | 3.79 | 0.0435 | TNFRSF1A,IL2RA,IL6 |
| IL-15 Signaling | 3.66 | 0.0395 | CXCL8,IL6,FASLG |
| Growth Hormone Signaling | 3.58 | 0.037 | IGF2,IGF1,A2M |
| Altered T Cell and B Cell Signaling in Rheumatoid Arthritis | 3.55 | 0.0361 | SPP1,IL6,FASLG |
| IL-17 Signaling | 3.52 | 0.0353 | CXCL8,CRP,IL6 |
| Bladder Cancer Signaling | 3.5 | 0.0349 | CXCL8,ERBB2,EGFR |
| Crosstalk between Dendritic Cells and Natural Killer Cells | 3.46 | 0.0337 | TNFSF10,IL6,FASLG |
| Death Receptor Signaling | 3.43 | 0.033 | TNFRSF1A,TNFSF10,FASLG |
| Role of Osteoblasts, Osteoclasts and Chondrocytes in Rheumatoid Arthritis | 3.4 | 0.0176 | SPP1,IGF1,TNFRSF1A,IL6 |
| IGF-1 Signaling | 3.24 | 0.0283 | IGF1,IGFBP5,IGFBP7 |
| Glioma Signaling | 3.2 | 0.0275 | IGF2,IGF1,EGFR |
| IL-17A Signaling in Gastric Cells | 3.17 | 0.08 | CXCL8,EGFR |
| Role of Tissue Factor in Cancer | 3.09 | 0.0252 | CXCL8,FGA,EGFR |
| Intrinsic Prothrombin Activation Pathway | 3.07 | 0.0714 | KLK3,FGA |
