# Supplementary material for: The serum-based VeriStrat® test is associated with proinflammatory reactants and clinical outcome in non-small cell lung cancer patients
Source: BMC Cancer. 2018 Mar 20;18:310. doi: 10.1186/s12885-018-4193-0 (PMC5861613; doi:10.1186/s12885-018-4193-0)
Supplement: Supplementary file 9 — Table S7. Gene set enrichment analysis results based on biomarker association with VeriStrat status. (DOCX 18 kb) [file 12885_2018_4193_MOESM9_ESM.docx]

**Table S7 Gene set enrichment analysis results based on biomarker association with VeriStrat status.** UniProt IDs were manually assigned and used to map to Gene Ontology (GO) terms, as defined at geneontology.org., with the gene set enrichment analysis performed as outlined in Subramanian et al (Proc Natl Acad Sci U S A. 2005 Oct 25;102(43):15545-50). No corrections were made for multiple testing.

| **Enrichment Score** | | **p-value** |
| --- | --- | --- |
| MAPK [GO:0000187] [GO:0000186] | 0.2742 | 0.7450 |
| Acute phase response [GO:0006953] | 0.8125 | 0.0088 |
| angiogenesis [GO:0001525] | 0.3439 | 0.6169 |
| Apoptosis [GO:0006915] [GO:0097190] | -0.4139 | 0.5565 |
| cell proliferation [GO:0008283] | -0.3884 | 0.4265 |
| chemoattractant activity [GO:0042056] | 0.4753 | 0.3476 |
| epidermal growth factor receptor signaling pathway [GO:0007173] | -0.3801 | 0.4979 |
| G-protein coupled receptor signaling pathway [GO:0007186] | 0.3436 | 0.8511 |
| immune response [GO:0006955] | 0.2325 | 0.9412 |
| inflammatory response [GO:0006954] | 0.5556 | 0.0971 |
| innate immune response [GO:0045087] | -0.2798 | 0.7070 |
| MAPK cascade [GO:0000165] | -0.3369 | 0.5864 |
| Necrosis [GO:0070266] [GO:0097527] | -0.8622 | 0.0304 |
| negative regulation of apoptotic process [GO:0043066] | -0.3057 | 0.6513 |
| positive chemotaxis [GO:0050918] | 0.4885 | 0.2579 |
| positive regulation of angiogenesis [GO:0045766] | 0.2232 | 0.9404 |
| positive regulation of ERK1 and ERK2 cascade [GO:0070374] | 0.4017 | 0.3154 |
| positive regulation of I-kappaB kinase/NF-kappaB signaling [GO:0043123] | -0.5441 | 0.4407 |
| MAPK cascade [GO:0043406] + [GO:0043410] | -0.3658 | 0.3085 |
| protein tyrosine kinase activity [GO:0004713] | -0.6769 | 0.1553 |
| Ras protein signal transduction [GO:0007265] | -0.4508 | 0.3036 |
| transmembrane receptor protein tyrosine kinase activity [GO:0004714] | -0.6190 | 0.1852 |
| vascular endothelial growth factor receptor signaling pathway [GO:0048010] | -0.2638 | 0.7754 |
| wound healing [GO:0042060] | -0.3548 | 0.8193 |
| RAS/RAF/MEK/ERK signaling [GO:0000186]+[GO:0007265]+[GO:0070374]+ [GO:0043406]+[GO:0043410]+[GO:0000165] | -0.2464 | 0.7505 |
| Angiogenesis [GO:0001525]+[GO:0048010]+ [GO:0045766] | -0.2093 | 0.9125 |
| P13K/AKT/Apoptosis-related [GO:0043066]+[GO:0051897]+[GO:0014068] | -0.3117 | 0.5422 |
| Growth Factor Signaling [GO:0007166]+[GO:0005125]+[GO:0007173]+[GO:0008083]+[GO:0007165]+[GO:0008286] | -0.2731 | 0.5887 |
| Metabolic [GO:0044267] | 0.3649 | 0.3263 |
| Coagulation/Response to Wounding [GO:0007596]+[GO:0030168]+[GO:0030168]+[GO:0002576] | 0.3085 | 0.6256 |
| Immune/Inflammatory [GO:0006955]+[GO:0006954]+GO:0045087]+[GO:0006953] | 0.3349 | 0.3805 |
| positive regulation of cell migration [GO:0030335] | -0.4626 | 0.2770 |
| positive regulation of cell proliferation [GO:0008284] | -0.3223 | 0.5012 |
| response to hypoxia [GO:0001666] | -0.1793 | 0.9946 |
| small GTPase mediated signal transduction [GO:0007264] | -0.3739 | 0.4614 |
| cell proliferation [GO:0008283] | -0.3884 | 0.4265 |
